# Exosomal MicroRNAs in Breast Cancer towards Diagnostic and Therapeutic Applications

**DOI:** 10.3390/cancers9070071

**Published:** 2017-06-24

**Authors:** Lorenzo F. Sempere, Jessica Keto, Muller Fabbri

**Affiliations:** 1 Center for Cancer and Cell Biology, Van Andel Research Institute, Grand Rapids, MI 49503, USA; 2 Comprehensive Breast Center, Mercy Health Saint Mary’s, Grand Rapids, MI 49503, USA; ketojl@mercyhealth.com; 3 Department of Pediatrics and Microbiology & Molecular Immunology, University of Southern California-Keck School of Medicine Norris Comprehensive, Cancer Center Children’s Center for Cancer and Blood Diseases, Children’s Hospital, Los Angeles, CA 90027, USA; mfabbri@chla.usc.edu

**Keywords:** microRNA, miRNA, miR, exosome, exosomal, extracellular vesicle, breast cancer, serum, plasma, blood

## Abstract

Soon after the discovery of microRNAs over 15 years ago, a myriad of research groups around the world sought to develop clinical applications in breast cancer for these short, noncoding, regulatory RNAs. While little of this knowledge has translated into the clinic, the recent research explosion on cell-to-cell communication via exosomes and other extracellular vesicles has rekindled interest in microRNA-based clinical applications. microRNAs appear to be a preferential and important cargo of exosomes in mediating biological effects in recipient cells. This review highlights recent studies on the biology of exosomal microRNAs (exo-miRNAs) and discusses potential clinical applications. From a diagnostic perspective, circulating exo-miRNAs may represent breast cancer cell content and/or tumor microenvironmental reactions to cancer cell growth. Thus, serum or plasma analysis of exo-miRNAs could be useful for early disease detection or for monitoring treatment response and disease progression. From a therapeutic perspective, exo-miRNAs derived from different cell types have been implicated in supporting or restraining tumor growth, conferring drug resistance, and preparing the metastatic niche. Strategies to interfere with the loading or delivery of tumor-promoting exo-miRNAs or to replenish tumor-suppressive miRNAs via exosomal delivery are under investigation. These recent studies provide new hope and opportunities, but study design limitations and technical challenges will need to be overcome before seriously considering clinical application of exo-miRNAs.

## 1. Introduction

Breast cancer is the most common type of cancer in women worldwide, with an estimated 1.7 million cases diagnosed in 2012 [1]. Despite significant advances in early detection and treatment, breast cancer remains the second-leading cause of cancer-related deaths, with an estimated 40,160 deaths in the U.S. in 2017 [2]. Further, an estimated 3.3 million U.S. women were living with breast cancer in 2014 [3]. These numbers highlight the ongoing challenges in the treatment of breast cancer, and specifically in developing strategies to prevent distant recurrence and to control disease in the metastatic setting. Tumors as small as 6 mm, i.e., near the resolution of imaging capabilities, can warrant adjuvant systemic therapy with associated toxicities due to the metastatic potential of aggressive tumor subtypes including triple-negative and HER2+ breast cancers. In addition, distant recurrence continues to be a short-term challenge in these aggressive subtypes, as does the late recurrence of estrogen-driven cancers. Strategies and challenges to reduce metastatic disease include early disease detection, enhancing treatment efficacy in the primary tumor to limit residual disease or metastatic seeding, and/or promotion of cell dormancy in metastatic niches. This review focuses on the potential use of microRNAs in exosomes to address these clinical needs.

Exosomes are membrane-bound vesicles, 50–200 nm in size, secreted from cells via a multivesicular-body endocytic process [4]. All cell types are thought to secrete exosomes, but the main functions of exosomes remain to be fully understood. Depending on context and cell type, exosomes have been proposed to be (1) a mechanism to eliminate content (DNA, RNA, or protein) detrimental to cell viability or fitness; (2) a cell-to-cell communication mechanism by delivering cargo to a recipient cell or providing ligand-receptor interactions on the plasma membrane of a recipient cell; and/or (3) a mechanism for surveying cell content for viral infections For example, in HER2+ breast cancers, HER2-expressing exosomes can serve as a decoy, interfering with the binding of a therapeutic antibody to HER2-expressing cancer cells and consequently lowering treatment efficacy [5,6].

While most researchers would agree on the definition of exosome based on their intracellular origin from an endocytic process and their size, many would disagree about the best method for obtaining highly pure exosomes. Recent reviews describe in more detail the advantages and limitations of different purification methods [7,8]. Differential ultracentrifugation or commercial kits such as exoQuick are among the most common methods for isolating exosomes from serum or plasma. Recently, the International Society for Extracellular Vesicles published a consensus document on the minimal experimental requirements for the characterization of exosomes [9]. Here we use the term “exosome” in a looser sense for convenience. For this review, we include studies that report on microRNAs in exosomes or in extracellular vesicles such as microvesicles budding from plasma membrane [10]. We rely on authors’ self-reporting of their definition and of the purity of their isolated extracellular vesicles as exosomes. We acknowledge that the studies we review used different methodologies and analyses, and that in several of the studies, the term “exosome” may reflect a mixture of different extracellular vesicles and large RNA-protein complexes (e.g., Ago-2) [11,12]. We conducted a systematic PubMed search for the terms breast cancer, exosome, extracellular vesicle, and microRNA. The focus of this review is the primary literature published between April 2014 and April 2017. The reader is referred to recent reviews for earlier studies.

microRNAs (miRNAs) are short, noncoding, regulatory RNAs that modulate gene expression at the post-transcriptional level, mostly via binding to partially complementary sites at the 3′ UTR of target mRNAs [13]. The first miRNA expression profiling studies on breast cancer tissue [14,15] date back to 2005. There are now over 2000 publications on PubMed reporting on different aspects of miRNA expression and function in breast cancer specimens and cell lines [16]. While some of these studies suggested cell-extrinsic effects of miRNA via regulation of secreted factors [17], the discovery of miRNAs as an abundant cargo in exosomes [16] suggested the possibility of direct, miRNA-mediated regulation in recipient cells both near to or distant from the donor cell expressing the miRNA(s). Follow-up studies [18,19,20] solidified the concept that miRNAs are an important and preferential cargo of exosomes in mediating biological effects in recipient cells [21,22,23]. There are now over 60 publications on PubMed related to the diagnostic and therapeutic potential of exosomal miRNAs (exo-miRNAs) in breast cancer. We discuss many of these studies in the following sections.

## 2. Detection of Circulating Exo-miRNAs and Their Diagnostics Implications

Cancer patients have a larger number of circulating exosomes relative to healthy controls [4]. This suggests that circulating exo-miRNAs in cancer patients disproportionally represent exosomes derived from cancer cells and/or other cell types in the tumor microenvironment (TME) rather than from non-involved normal cells. Thus, detection of exo-miRNAs could be used for diagnostic or prognostic purposes in breast cancer [8].

### 2.1. Circulating Exo-miRNAs in Serum or Plasma for Early Disease Detection

A reliable blood test that complements the sensitivity and specificity of mammography screening would be an ideal tool for early detection. There have been many studies on detecting circulating miRNAs in the serum or plasma of breast cancer patients vs. healthy controls. Unfortunately, there is relatively little overlap between identified signatures [16]. Exo-miRNAs are thought to be the predominant source of circulating miRNAs isolated from plasma or serum, based on comparing matching exosome and tissue samples or matching exosome and cell line samples [24]. An emerging idea is that detecting miRNAs in the exosome fraction isolated from plasma or serum can provide a higher quality and more consistent readout than “crude” examination of plasma or serum samples [25,26,27]. Currently, there are a few studies that have analyzed circulating exo-miRNAs from breast cancer patients. In a study of 50 breast cancer cases and 12 healthy controls with matched serum and exosomes, the levels of miR-101 and miR-372 were significantly higher in cancer cases when detecting these miRNAs in RNA isolated from exosomes, but not in serum RNA preparations [28]. Conversely, the levels of miR-373 were significantly higher in cancer cases when detecting this miRNA in serum, but not in the exosome preparations [28]. Intriguingly, subgroup analysis based on estrogen (ER) or progesterone (PR) receptor status showed that exo-miR-373 levels are significantly higher in receptor-negative cases, consistent with higher levels of miR-373 in serum from triple-negative breast cancer (TNBC) cases [28]. The differential expression of exo-miRNAs based on tumor subtype may be useful in identifying the most aggressive subtypes. Note that serum analysis of these three miRNAs (miR-101, miR-372, miR-373) did not separate benign breast conditions from breast cancer; exosomal analysis for these miRNAs to separate these two groups was not reported. In another study, exo-miR-21 and exo-miR-1246 were consistently detected at higher levels in the plasma of mice growing patient-derived tumors or in the plasma of breast cancer patients, relative to healthy control groups [29]. These findings agree with previous studies that showed high levels of these miRNAs in serum or plasma [30,31,32]. Analysis of exo-miRNAs from conditioned media of breast cancer cell lines and immortalized breast epithelial cells suggest a differential loading of some miRNAs in exosomes and support the idea that the circulating levels may be higher than in the originating cells [25,26,33,34]. Such exo-miRNAs could be good candidates for early disease detection. Indeed, miR-1246 was among the top miRNAs selectively loaded and secreted in exosomes derived from breast cancer cells (MCF7, Sk-Br-3, MDA-MB-231) relative to immortalized cell lines (MCF10A, IRM90) [25]. Exo-miR-182 levels were significantly higher than those of exo-miR-96 and exo-miR-183, but these miRNAs were expressed at similar levels in originating breast cancer cells (MCF7, BT474, MDA-MB-231) [34]. A concern with cell culture studies is that fetal bovine serum (FBS), which is used in most media preparation can contain exo-miRNAs or free-circulating miRNAs, identical in sequence to human miRNAs, and confound result interpretation [35]. miR-122, miR-451a and miR-1246 are abundant in FBS and reported enrichment of these miRNAs in exosome may, at least in part, have a bovine origin [35].

### 2.2. Circulating Exo-miRNAs to Monitor Treatment Response

There have been several studies analyzing circulating miRNA levels in serum or plasma from patients before and after neoadjuvant treatment. Differences in drug treatments and tumor subtypes in those studies have hampered identification of a robust signature [16]. We found no reports focused on exo-miRNAs for predicting or monitoring tumor response in blood samples from patients undergoing treatment. Exo-miR-503 derived from endothelial cells can be transferred to breast cancer cell lines in vitro, and they modify the cells’ proliferation rate and invasive phenotype [36]. Circulating levels of miR-503 were increased in patients undergoing neoadjuvant treatment. These results suggest at least some contribution from endothelial-derived exo-miR-503, but the authors did not directly test this hypothesis [36]. In the next section, we discuss exo-miRNAs from breast cancer cells lines that modulate resistance to specific chemotherapy drugs, which might provide candidates for monitoring treatment response in patients (Table 1).

### 2.3. Limitations, Challenges and Opportunities for Diagnostic Application

There are few studies focused on exo-miRNA detection in breast cancer. More studies are needed to assess the robustness and reproducibility of exo-miRNAs and to independently validate exo-miRNA signatures. Harmonization of technology for exosome isolation and exo-miRNA detection will be important in order to directly compare results across studies. Currently, size-exclusion chromatography is considered the most reliable method for the most highly enriched isolation of exosomes, but current protocols will be difficult to implement in clinical setting [7]. The field as a whole may need to come to a compromise between a technology that allows for a consistent enrichment of exosomes with an acceptable amount of other “contaminating” extracellular vesicles and a technology that can be implemented in a clinical laboratory [59].

Exo-miRNA detection also offers unique analysis opportunities. Proteins displayed on the exosome surface match those of the plasma membrane of source cells. Antibody-based selection of cell type–specific surface proteins could be used to enrich and interrogate with less noise the content of a specific cell type in a tumor. Epithelial Cell Adhesion Molecule (EpCam)-based capturing of exosomes for miRNA detection has been reported for colorectal cancer [60], and there are some concerns about the loss of EpCam presence in epithelial cell–derived cancer cells because of no (or low) expression or cleavage of EpCam [61]. It will be important to test whether exo-miRNA analysis in EpCam+ exosomes outperforms other EpCam-based assays for early disease detection and disease monitoring (such as for the number of circulating tumor cells). Similarly, it will be important to test whether exo-miRNAs in preparations enriched for exosomes derived from tumor-associated fibroblasts or other cell types provide additional information to EpCam-selected exosome analysis.

## 3. Functions of Exo-miRNAs and Their Therapeutic Implications

Functional studies in breast cancer cell lines, co-cultures, and xenograft models have unraveled the roles of miRNAs in cancer cells or other cell types of the tumor microenvironment (TME) in supporting or restraining tumor growth, conferring drug resistance, and preparing the metastatic niche. miRNA-mediated regulation of one cell type could affect other cell types in the TME by regulating the levels of secreted ligands, cytokines, and chemokines [17]. Exo-miR-21 released by cancer cells can be taken up by tumor-associated macrophages (TAMs) within the TME [62]. TAMs express Toll-like receptor 8 (TLR8), the first identified receptor for miRNAs, an “miRceptor”. By binding to TLR8, exo-miR-21 activates NF-κB signaling in TAMs, leading to transactivation of miR-155, which is then released as an exo-miRNA and is shuttled back to cancer cells, where it increases resistance to cisplatin by directly targeting TERF1 [63]. While this mechanism has been described in detail in neuroblastoma, co-culture of cancer cells (including breast cancer cells) and human monocytes in a Transwell system (no cell-to-cell contact) consistently results in increased levels of miR-155 and reduced levels of TERF1 in the recipient cancer cell. These data suggest that the described mechanism (the first of its kind showing the ability of exo-miRNAs to bind to and activate a receptor signaling) may be involved in the acquisition of drug resistance in a variety of human cancers [64], including breast cancer [63]. The facts that (1) elevated levels of selective miRNAs are found in exosomes from breast cancer patients and (2) exosomes derived from breast cancer patients induce the transformation of nonmalignant breast epithelial cells in a Dicer-dependent manner [65,66] suggest the involvement of exo-miRNAs in transferring functions or characteristics of a donor cell into a recipient cell. 

### 3.1. Cancer Cell-Derived Exo-miRNAs Modulate Resistance to Hormone Therapy

Tamoxifen and aromatase inhibitors are the main anti-hormonal treatments for ER+ tumors. Expression profiling in human breast cancer tissues and ER+ cell lines have identified many miRNAs that correlate with ER status and some miRNAs that can confer resistance to tamoxifen treatment, including miR-221 and miR-222. Previous studies indicated that miR-221 and miR-222 down-regulate ERα and p27 [67]. A study suggests that exo-miR-221 and exo-miR-222 can be transferred from tamoxifen-resistant MCF7 to sensitive MCF7 cells and can increase the resistance to tamoxifen in recipient cells via regulation of these two targets [37]. It would be interesting to see if other miRNAs linked to anti-hormonal resistance (such as miR-342 and miR-519a) also confer resistance to hormonal treatment via exosomal transfer [16,68].

### 3.2. Cancer Cell-Derived Exo-miRNAs Modulate Resistance to Chemotherapy Agents

A combination of anthracyclines, cyclophosphamide, and/or taxanes are the backbone of most chemotherapy regimens for breast cancer treatment. Several miRNAs (including miR-21, miR-29a, miR-100, miR-221, and miR-222) have been proven to modulate chemoresistance in breast cancer cells to chemotherapy drugs, including doxorubicin and docetaxel [69,70]. While these studies focused on cancer-cell-intrinsic changes of miRNA expression, recent studies have uncovered roles for the same miRNAs in exosomes in contributing to chemoresistance (Table 1). Exosomes derived from adriamycin-resistant or docetaxel-resistant MCF7 cells can confer chemoresistance to sensitive MCF7 cells. miR-17, miR-30a, miR-100, and miR-222 are up-regulated in MCF7 cells after drug treatment and are enriched in exosomes derived from treated cells [38]. These exo-miRNAs are thought to mediate, at least in part, chemoresistant effects.

Transfection of miR-29a and/or miR-222 mimetic RNAs into parental MCF7 cells increases resistance to either adriamycin or docetaxel, whereas transfection of miR-222 inhibitor increases sensitivity to these drugs [39,41,42,43,71]. These two miRNAs down-regulate PTEN expression, which may contribute to the observed phenotype. These results suggest that the delivery of miR-29- and/or miR-222-enriched exosomes enhances resistance to these chemotherapy agents in the recipient cells. However, this possibility was not directly tested. Another study analyzed changes of exo-miRNA levels in MDA-MB-231 sub-lines that developed resistance to docetaxel, epirubicin, or vinorelbine [33]. Ten miRNAs were up-regulated in cells and were also loaded at increased copy numbers in exosomes from docetaxel-resistant MDA-MB-231 cells; 11 miRNAs from epirubicin-resistant MDA-MB-231 cells; and 4 from vinorelbine-resistant MDA-MB-231 cells (Table 1). None of these miRNAs were represented in all signatures, and only two common miRNAs (miR-138-5p, miR-210-3p) were present in the epirubicin- and vinorelbine-resistant signatures. The functional contribution of these exo-miRNAs was not investigated.

Others studies have teased out specific contributions of individual exo-miRNAs in modulating chemoresistance and other cell properties. The delivery of miR-134-enriched exosomes into the TNBC cell line Hs578Ts(i)8 reduced its cell migration and invasiveness and increased its sensitivity to a drug targeting Hsp90 [47]. Down-regulation of STAT5B and Hsp90 protein expression via exo-miR-134, although not as profound as that due to lipofectamine-mediated transfection, was proposed as a main molecular mechanism for the observed phenotypes [47]. The restoration of miR-134 activity via transfection enhanced the cisplatin-mediated apoptotic rates, but these chemosensitivity effects were not studied with exo-miR-134 supplementation.

### 3.3. Cancer Cell-Derived Exo-miRNAs Promote Invasion and Metastasis

Members of the miR-200 family are master regulators of maintenance of epithelial programs by down-regulating ZEB1/2 transcriptional repressors of E-cadherin and other epithelial genes, including miR-200 family members themselves. The miR-200 family members maintain epithelial differentiation, suppress the epithelial-to-mesenchymal (E-MT) transition, and also enhance the mesenchymal-to-epithelial transition (M-ET). miR-200 family members have been shown to facilitate metastasis formation by inducing an M-ET for the growth in distant sites of circulating tumor cells having an E-MT phenotype or by maintaining an epithelial phenotype in cancer cells that colonize without undergoing E-MT [72]. In syngeneic mouse models using different experimental approaches, exo-miR-200 from highly metastatic cell lines that express large amounts of members of the miR-200 family enhanced the metastatic potential of nonmetastatic cancer cells that do not express those miRNAs [49]. Tail vein injection of nonmetastatic 4T07 cells incubated with exosomes from the highly metastatic 4T1 epithelial subline (which expresses high levels of miR-200s) significantly increased the incidence of lung metastasis. Remarkably, small primary tumors that express miR-200s but have no metastatic potential themselves were able to confer metastatic potential to tail-vein injected 4T07 cells via exo-miR-200s. In some breast cancer studies, miR-10b expression correlated with the incidence of metastasis, and the forced expression of miR-10b in nonmetastatic breast cancer cell lines enhanced their metastatic potential [73]. Exo-miR-10b secreted from metastatic MDA-MB-231 cells increased the invasion ability of nonmalignant immortalized human mammary epithelial cells (HLME) via down-regulation of the known targets HoxD10 and KLF4 [48].

While these cancer-cell-derived exo-miRNAs appear to predominantly transfer functions to other pre-malignant or malignant epithelial cells, other exo-miRNAs are transferred into noncancerous recipient cells to promote metastasis. miR-9 is considered a pro-metastatic miRNA that regulates E-cadherin expression within cancer cells. Exo-miR-9 transfer to normal fibroblasts can enhance their conversion to tumor-associated fibroblasts (TAFs) to support cancer cell growth [50]. TAFs of aggressive TNBC cases expressed higher levels of miR-9 than those other subtypes [50]. TAF-derived exo-miR-9 can be transferred to cancer cells, increasing miR-9 expression and down-regulating E-cadherin expression. Exo-miR-105 is taken up by endothelial cells in which it down-regulates tight junction protein ZO-1 [52]. This leads to increased vascular permeability and extravasation of breast cancer cells to distant organs [52]. Similarly, exo-miR-181c compromises the blood–brain barrier and facilitates seeding of breast cancer cells to the brain [53]. Exo-miR-181c targets 3-phosphoinositide-dependent protein kinase-1 (PDPK1) in endothelial cells which leads to abnormal actin dynamics and fiber arrangements, and the weakening of tight junctions [53]. Exo-miR-122 can reprogram glucose metabolism of noncancerous cells in the pre-metastatic niche including fibroblasts in the lung and astrocytes and neurons in the brain to free up nutrients for incoming cancer cells [51]. Both in vitro and in vivo delivery of exo-miR-122 down-regulated expression of pyruvate kinase in noncancerous recipient cells and decreased their glucose utilization [51].

### 3.4. Cancer Cell- and Mesenchymal Stem Cell-Derived Exosomes Modulate Angiogenesis

Docosahexaenoic acid (DHA) is a natural compound with anti-angiogenic properties, which is under investigation as dietary supplement for breast cancer prevention and treatment. In vitro treatment with DHA alters exosome secretion rate and miRNA cargo of MCF7 and other breast cancer cell lines [54]. The most pronounced changes were noted for exo-let-7s, exo-miR-21, exo-miR-23a, exo-miR-27a/b, and exo-miR-320 [54]. Exosomes derived from DHA-treated MCF7 cells increased expression of these miRNAs in the recipient EA.hy926 endothelial cell line and reduced tubular formation. Transfection of miR-23b and miR-320b mimetic compound into EA.hy926 cells reduced tubular formation and down-regulated expression of their pro-angiogenic target genes (PLAU, AMOTL1, NRP1, and ETS2) [54]. Exosomes derived from mesenchymal stem cells (MSCs) in the TME can also exert anti-angiogenic effects by down-regulating (Vascular Endothelial Growth Factor (VEGF) expression in recipient cancer cells. Exo-miR-16 is enriched in MSC-derived exosomes and is thought to contribute in part in this anti-angiogenic mechanism as it is known to directly regulate VEGF expression [54]. 

### 3.5. Stromal Cell-Derived Exo-miRNAs Modulate Cancer Stem Cell-like Properties

Interactions between preadipocytes and cancer cells promote tumor growth and metastatic spread. Exosomes derived from preadipocytes contribute to cancer stem-like cell niche formation. Co-culture experiments with mouse preadipocyte 3T3L1 cell and MCF10DCIS as a model of early-stage breast cancer identified preadipocyte-derived exo-miR-140 as an important negative regulator of cancer stem-like cell renewal and cell migration via targeting of SOX9 [46]. Treatment of 3T3L1 cells with Chinese herbal medicine shikonin (a naphthoquinone) increased exosomal secretion of miR-140 [46]. Co-culture of MCF10DCIS with exosomes derived from shikonin-treated 3T3L1 inhibited growth, whereas exosomes derived from untreated cells promoted breast cancer cell growth in an in vivo model [46]. Shikonin is known to have many biological activities ranging from antiviral to anti-angiogenic. Regulation of miRNA cargo in exosomes appears to be another and complex biological activity of shikonin. While shikonin treatment of 3T3L1 results in preferential loading of miR-140 in exosomes, treatment in MCF7 reduces exosomal loading of pro-growth and anti-apoptotic miR-128 [44]. Exosomes derived from TAFs also modulate proliferation, stem cell-like capacity and E-MT of cancer cells. Exo-miR-21, exo-miR-143, and exo-miR-378e are enriched in exosomes derived from TAFs compared to normal fibroblasts [45]. Co-culture of TAF-derived exosomes with BT549, MDA-MB-231 or T47D increased anchorage-dependent and anchorage-independent growth of these breast cancer cell lines, which also exhibit increased expression of stem-cell like and mesenchymal markers [45]. Transfection of normal fibroblasts with miR-21, miR-143 and miR-378e mimetics, prior to co-culture of fibroblast-derived exosomes with cancer cells, resulted in similar enhanced malignant phenotypes in breast cancer cells, suggesting a functional role for these three TAF-derived exo-miRNAs [45]. 

### 3.6. Stromal Cell-Secreted Exo-miRNAs Modulate Cancer Cell Dormancy

Bone marrow metastasis can develop decades after initial diagnosis. Stromal cells, including MSCs, in the bone microenvironment interact with disseminated cancer cells via physical contact, paracrine signaling and exosome transfer. Bone marrow stroma can induce cancer cell quiescence and long-term dormancy. Co-culture of bone marrow stroma with MDA-MB-231 or T47D breast cancer cells induced G0-cell cycle arrest [57]. Bone marrow stromal cell–derived exo-miR-127, exo-miR-197, exo-miR-222, and exo-miR-223 are taken up by T47D cells in which these miRNAs repress CXCL12 expression [57]. CXCL12 is a chemokine that interacts with CXR4 and CXR7 receptors. Loss of CXCL12 in cancer cells disrupts their physical interaction with CXR4-expressing hematopoietic stem cells creating a more hostile microenvironment for this growth. While this seems to be a tumor-suppressive mechanism to restrain metastatic growth, cancer cells entering quiescence may acquire the selective advantage of being more resistant to chemotherapy [58]. MSC-derived exosomes from naïve cells (not exposed or interacting with cancer cells) do not induce G0-cell cycle arrest, only exosomes from primed MSCs (exposed or in contact with cancer cells) induced cell cycle arrest [58] as originally reported [57]. Exo-miR-222 and exo-miR-223 are 5-fold enriched in exosomes from primed MSCs compared to naïve MSCs and confer resistance to breast cancer cell lines against carboplatin treatment. In an in vivo femur model of breast cancer dormancy, transfection of MSCs with anti-miR-222/223 inhibitors before bone implantation sensitized breast cancer cells to low-dose carboplatin treatment by preventing the transfer of MSC-derived exo-miR-222/223 and/or by delivering anti-miR-222/223 in exosomes to the cancer cells [58]. This study did not investigate which factor(s) from the cancer cells were triggering this priming. Exo-miR-23b derived from MSC can also induce dormancy and increase chemoresistance to docetaxel. Co-culture of BM2 (an MDA- MB-231 subline with increased propensity to form bone marrow metastasis) and MSCs (obtained from human donors) suppressed proliferation, decreased stem-cell like properties, and migratory capability of BM2 cells [56]. Exo-miR-23b was among the most enriched miRNAs in the cargo of MSC-derived exosomes compared to exosomes from adult fibroblasts. The myristoylated alanine-rich C kinase substrate (MARCKS) was identified as a key target of exo-miR-23b in recipient BM2 cells [56]. miR-23b expression was increased in metastatic bone lesions compared to matched primary breast tumors from a cohort of 10 patients, whereas MARCKS expression was decreased [56]. This provides support to the clinical relevance of miR-23b-mediated regulation of dormancy. 

### 3.7. Limitations with Current Study Designs and Perceived Challenges for Therapeutic Application

Most reports mechanistically dissecting the role of exo-miRNA have been conducted in in vitro cell line systems or co-culture systems in which conditioned media containing exosomes or purified exosome preparations have been incubated with intended recipient cells. In many cases, it is not clearly established whether the amount of exo-miRNAs and other cargo delivered by this approach is within a physiological range. The amount of miRNAs in exosomes is typically several orders of magnitude below cellular levels [33], one recent study suggests a typical exosome may carry just a single molecule of any given miRNA [74]. Thus, exogenous supplementation of native and synthetic exosomes may exaggerate the function of exo-miRNA cargo, and it may be difficult to extrapolate the effects of these in vitro manipulations to an in vivo system, in which it would be more challenging to track transfer of exosomes from donor to a specific recipient cell [75]. Most studies use a limited number of well-established cell lines, mainly, MCF7 and/or T47D as ER+ models and MDA-MB-231 as a TNBC model. Besides the hormonal status, it must be pointed out that genetic mutations, epigenetic alterations, and global miRNA profiles are different between MCF7 and T47D compared to MDA-MB-231. Thus, it is interesting that there was no obvious overlapping of increased exo-miRNA levels between docetaxel-resistant MCF7 and docetaxel-resistant MDA-MB-231 cells in chemoresistance studies, while in other studies on selective exosomal loading and dormancy there were more similarities in the exo-miRNAs content and function between MCF7 (and/or T47D) and MDA-MB-231 (and other TNBC cell lines). Collectively, these studies support the idea that specific exo-miRNAs derived from breast cancer cells have a more profound effect in nonmalignant epithelial cells, MSC, endothelial cells or other stromal cells. Likewise, specific exo-miRNAs derived from MSC, endothelial cells and other stromal cells have a profound effect in cancer cells. It is a bit puzzling and a challenge in the field to envision how donor cells manage to exquisitely load and sort differential miRNA cargo in distinct subsets of exosomes with differential affinity towards distinct recipient cells. Several efforts have been conducted to better understand the miRNA sorting mechanism to exosomes, leading to the remarkable conclusion that exosome enrichment is modulated by cell-activation-dependent changes of miRNA target levels in the donor cells [76]. Integrins decorating exosomes’ membranes are important determinants for selective delivery of cancer cell-derived exosomes to different distant organs, with integrin α_6_β_4_- and α_6_β_1_-decorated exosomes favoring delivery to the lung and α_v_β_5_ to the liver [77]. Other surface proteins yet to be characterized may also contribute to the selective delivery of exosomes.

## 4. Conclusions

We are still in the early days of exosomal research in breast cancer. From a diagnostic perspective, it is likely that studies on circulating exo-miRNA analysis in patient blood samples will flourish within the next few years. These studies will enable a more critical evaluation of the clinical value of exo-miRNAs in total or cell type-enriched exosomal preparations. From a therapeutic perspective, a major impediment continues to be the inability of nucleic acid-based miRNA activity modulators to reach breast cancer cells or cellular compartment of the tumor microenvironment. Strategies that target exosomes rather than cancer cells may provide new opportunities to inhibit tumor-promoting miRNAs. These strategies may include transfection of anti-miRNA compounds into exosomes, drugs that selectively interfere with loading or delivery of tumor-promoting exo-miRNAs, or depletion of specific exosome subsets from circulation. Native or synthetic exosomes with cell-type-specific affinities could be harnessed to deliver miRNA-activity-modulating compounds to metastatic cancer cells or other components of the microenvironment to replenish tumor-suppressive miRNAs or inhibit tumor-promoting miRNAs, respectively.

## Figures and Tables

**Table 1:** Functional studies of exo-miRNAs in breast cancer.

Exo-miRNA(s)	Donor cell	Recipient cell	Biological activity	Evidence	Gene target(s)	Experimental system	Refs
miR-221, miR-222	Cancer cell	Cancer cell	Hormonal resistance	Functional (exosome transfer)	ER, p27	in vitro (MCF7)	[37]
miR-17, miR-30a, miR-100, miR-222	Cancer cell	Cancer cell	Drug resistance (adriamycin, docetaxel)	Differential exosomal representation		in vitro (MCF7)	[38]
miR-29a, miR-30a, miR-100, miR-196a, miR-222	Cancer cell	Cancer cell	Drug resistance (adriamycin, docetaxel)	Differential exosomal representation		in vitro (MCF7)	[39]
miR-20a, miR-23a, miR-24, miR-149, miR-222	Cancer cell	Cancer cell	Drug resistance (adriamycin)	Increased levels in recipient cells suggestive of exosomal transfer		in vitro (MCF7)	[40]
miR-29a	Cancer cell	Cancer cell	Drug resistance (adriamycin)	Functional (transfection), differential exosomal representation	PTEN	in vitro (MCF7)	[41]
miR-29a, miR-222	Cancer cell	Cancer cell	Drug resistance (adriamycin, docetaxel)	Functional (transfection)	PTEN	in vitro (MCF7)	[39]
miR-222	Cancer cell	Cancer cell	Drug resistance (adriamycin)	Functional (transfection), differential exosomal representation	PTEN	in vitro (MCF7)	[42]
miR-222	Cancer cell	Cancer cell	Drug resistance (adriamycin)	Functional (transfection), differential exosomal representation		in vitro (MCF7)	[43]
miR-138-5p, miR-139-5p, miR-197-3p, miR-210-3p, miR-423-5p, miR-574-3p, miR-744-5p, miR-3178, miR-4258, miR-4443, miR-6780b-3p	Cancer cell	Cancer cell	Drug resistance (epirubicin)	Differential exosomal representation		in vitro (MDA-MB-231)	[33]
miR-138-5p, miR-140-3p, miR-210-3p, miR-3613-5p	Cancer cell	Cancer cell	Drug resistance (vinorelbine)	Differential exosomal representation		in vitro (MDA-MB-231)	[33]
miR-149-3p, miR-423-5p, miR-671-5p, miR-1246, miR-1268a, miR-4298, miR-4438, miR-4644, miR-7107-5p, miR-7847-3p	Cancer cell	Cancer cell	Drug resistance (docetaxel)	Differential exosomal representation		in vitro (MDA-MB-231)	[33]
miR-128	Cancer cell	Cancer cell	Proliferation	Functional (exosome transfer, shikonin)	BAX	in vitro (MCF7)	[44]
miR-21, miR-143, miR-378e	Fibroblast	Cancer cell	Proliferation, stem cell renewal, and invasion	Functional (exosome transfer, transfection)		in vitro (patient-derived fibroblasts, BT549, MDA-MB-231, T47D)	[45]
miR-140	Preadipocyte	Cancer cell	Proliferation, stem cell renewal	Functional (shikonin)	SOX9	in vitro, xenograft (3T3L1, MCF10DCIS)	[46]
miR-503	Endothelial cell	Cancer cell	Proliferation and invasion	Functional (exosome transfer)	CCND2, CCND3	in vitro (HUVEC, MDA-MB-231)	[36]
miR-134	Cancer cell	Cancer cell	Migration and invasion	Functional (exosome transfer)	STAT5B	in vitro (Hs578Ts(i)8	[47]
miR-10b	Cancer cell	Epithelial cell	Migration and invasion	Functional (exosome transfer)	HoxD10, KLF4	in vitro (MDA-MB-231, HMLE)	[48]
miR-141, miR-200a, miR-200b, miR-200c, miR-429	Cancer cell	Cancer cell	Metastatic potential	Functional (exosome transfer), differential exosomal representation		in vitro, in vivo allograft (4TO7, 4TO1E)	[49]
miR-9	Cancer cell	Fibroblast	Metastatic potential	Functional (exosome transfer), differential exosomal representation		in vitro (MDA-MB-231, MDA-MB-468, patient-derived fibroblasts)	[50]
miR-9	Fibroblast	Cancer cell	Metastatic potential	Functional (exosome transfer), differential exosomal representation	E-cadherin	in vitro, in vivo xenograft (fibroblasts, MDA-MB-231, MDA-MB-468)	[50]
miR-122	Cancer cell	Fibroblast, neurons, microglia	Metabolic reprogramming, metastatic niche	Functional (exosome transfer)	Pyruvate kinase (​PKM2), Citrate synthase (​CS)	in vitro, in vivo xenograft (MCF10DCIS.com, MDA-MB-231, murine brain cortical neurons, murine lung fibroblasts)	[51]
miR-105	Cancer cell	Endothelial cell	Vascular permeability, extravasation	Functional (exosome transfer)	ZO-1	in vitro, in vivo xenograft (MDA-MB-231, HMVECs)	[52]
miR-181c	Cancer cell	Endothelial cell	Vascular permeability, extravasation	Functional (exosome transfer)	PDPK1	in vitro (MDA-MB-231.D3H2LN, endothelial cells)	[53]
let-7, miR-21, miR-23a, miR-27a/b, miR-320	Cancer cell	Endothelial cell	Angiogenesis	Functional (exosome transfer, docosahexaenoic acid)		in vitro (MCF7, EA.hy926)	[54]
miR-23, miR-320	Cancer cell	Endothelial cell	Angiogenesis	Functional (transfection)	PLAU, AMOTL1, NRP1, ETS2	in vitro (MCF7, EA.hy926)	[54]
miR-16	MSC	Cancer cell	Angiogenesis	Functional (exosome transfer)	VEGF	in vitro, in vitro allograft (murine MSC, 4T1)	[55]
miR-23b	MSC	Cancer cell	Dormancy, drug resistance (docetaxel)	Functional (exosome transfer)	MARCKS	in vitro (MDA-MB-231.BM2, MSC)	[56]
miR-127, miR-197, miR-222, miR-223	MSC	Cancer cell	Dormancy	Functional (exosome transfer)	CXCL12	in vitro, in vivo xenograft (MSC, MDA-MB-231, T47D)	[57]
miR-222, miR-223	MSC	Cancer cell	Dormancy, drug resistance (carboplatin)	Functional (exosome transfer, transfection)		in vitro, in vivo xenograft (MSC, MDA-MB-231, T47D)	[58]

## References

[1] Ferlay J., Soerjomataram I., Dikshit R., Eser S., Mathers C., Rebelo M., Parkin D.M., Forman D., Bray F. (2015). Cancer incidence and mortality worldwide: Sources, methods and major patterns in GLOBOCAN 2012. Int. J. Cancer.

[2] Siegel R.L., Miller K.D., Jemal A. (2017). Cancer statistics, 2017. CA Cancer J. Clin..

[3] National Cancer Institute Cancer Stat Facts: Female Breast Cancer. https://seer.cancer.gov/statfacts/html/breast.html.

[4] Kalluri R. (2016). The biology and function of exosomes in cancer. J. Clin. Investig..

[5] Ciravolo V., Huber V., Ghedini G.C., Venturelli E., Bianchi F., Campiglio M., Morelli D., Villa A., Della Mina P., Menard S. (2012). Potential role of her2-overexpressing exosomes in countering trastuzumab-based therapy. J. Cell. Physiol..

[6] Marleau A.M., Chen C.S., Joyce J.A., Tullis R.H. (2012). Exosome removal as a therapeutic adjuvant in cancer. J. Transl. Med..

[7] Thind A., Wilson C. (2016). Exosomal mirnas as cancer biomarkers and therapeutic targets. J. Extracell. Vesicles.

[8] Joyce D.P., Kerin M.J., Dwyer R.M. (2016). Exosome-encapsulated micrornas as circulating biomarkers for breast cancer. Int. J. Cancer.

[9] Lotvall J., Hill A.F., Hochberg F., Buzas E.I., Di Vizio D., Gardiner C., Gho Y.S., Kurochkin I.V., Mathivanan S., Quesenberry P. (2014). Minimal experimental requirements for definition of extracellular vesicles and their functions: A position statement from the international society for extracellular vesicles. J. Extracell. Vesicles.

[10] Kowal J., Arras G., Colombo M., Jouve M., Morath J.P., Primdal-Bengtson B., Dingli F., Loew D., Tkach M., Thery C. (2016). Proteomic comparison defines novel markers to characterize heterogeneous populations of extracellular vesicle subtypes. Proc. Natl. Acad. Sci. USA.

[11] Arroyo J.D., Chevillet J.R., Kroh E.M., Ruf I.K., Pritchard C.C., Gibson D.F., Mitchell P.S., Bennett C.F., Pogosova-Agadjanyan E.L., Stirewalt D.L. (2011). Argonaute2 complexes carry a population of circulating micrornas independent of vesicles in human plasma. Proc. Natl. Acad. Sci. USA.

[12] Turchinovich A., Weiz L., Langheinz A., Burwinkel B. (2011). Characterization of extracellular circulating microrna. Nucleic Acids Res..

[13] Steinkraus B.R., Toegel M., Fulga T.A. (2016). Tiny giants of gene regulation: Experimental strategies for microrna functional studies. Wiley Interdiscip. Rev. Dev. Biol..

[14] Iorio M.V., Ferracin M., Liu C.G., Veronese A., Spizzo R., Sabbioni S., Magri E., Pedriali M., Fabbri M., Campiglio M. (2005). Microrna gene expression deregulation in human breast cancer. Cancer Res..

[15] Lu J., Getz G., Miska E.A., varez-Saavedra E., Lamb J., Peck D., Sweet-Cordero A., Ebert B.L., Mak R.H., Ferrando A.A. (2005). Microrna expression profiles classify human cancers. Nature.

[16] Graveel C.R., Calderone H.M., Westerhuis J.J., Winn M.E., Sempere L.F. (2015). Critical analysis of the potential for microrna biomarkers in breast cancer management. Breast Cancer (Dove Med Press).

[17] Pencheva N., Tavazoie S.F. (2013). Control of metastatic progression by microrna regulatory networks. Nat. Cell Biol..

[18] Pegtel D.M., Cosmopoulos K., Thorley-Lawson D.A., van Eijndhoven M.A., Hopmans E.S., Lindenberg J.L., de Gruijl T.D., Wurdinger T., Middeldorp J.M. (2010). Functional delivery of viral mirnas via exosomes. Proc. Natl. Acad. Sci. USA.

[19] Kosaka N., Iguchi H., Yoshioka Y., Takeshita F., Matsuki Y., Ochiya T. (2010). Secretory mechanisms and intercellular transfer of micrornas in living cells. J. Biol. Chem..

[20] Zhang Y., Liu D., Chen X., Li J., Li L., Bian Z., Sun F., Lu J., Yin Y., Cai X. (2010). Secreted monocytic mir-150 enhances targeted endothelial cell migration. Mol. Cell.

[21] Kosaka N., Yoshioka Y., Fujita Y., Ochiya T. (2016). Versatile roles of extracellular vesicles in cancer. J. Clin. Investig..

[22] Frediani J.N., Fabbri M. (2016). Essential role of mirnas in orchestrating the biology of the tumor microenvironment. Mol. Cancer.

[23] Fanini F., Fabbri M. (2017). Cancer-derived exosomic micrornas shape the immune system within the tumor microenvironment: State of the art. Semin. Cell Dev. Biol..

[24] Nedaeinia R., Manian M., Jazayeri M.H., Ranjbar M., Salehi R., Sharifi M., Mohaghegh F., Goli M., Jahednia S.H., Avan A. (2017). Circulating exosomes and exosomal micrornas as biomarkers in gastrointestinal cancer. Cancer Gene Ther..

[25] Pigati L., Yaddanapudi S.C., Iyengar R., Kim D.J., Hearn S.A., Danforth D., Hastings M.L., Duelli D.M. (2010). Selective release of microrna species from normal and malignant mammary epithelial cells. PLoS ONE.

[26] Palma J., Yaddanapudi S.C., Pigati L., Havens M.A., Jeong S., Weiner G.A., Weimer K.M., Stern B., Hastings M.L., Duelli D.M. (2012). Micrornas are exported from malignant cells in customized particles. Nucleic Acids Res..

[27] Jenjaroenpun P., Kremenska Y., Nair V.M., Kremenskoy M., Joseph B., Kurochkin I.V. (2013). Characterization of rna in exosomes secreted by human breast cancer cell lines using next-generation sequencing. PeerJ.

[28] Eichelser C., Stuckrath I., Muller V., Milde-Langosch K., Wikman H., Pantel K., Schwarzenbach H. (2014). Increased serum levels of circulating exosomal microrna-373 in receptor-negative breast cancer patients. Oncotarget.

[29] Hannafon B.N., Trigoso Y.D., Calloway C.L., Zhao Y.D., Lum D.H., Welm A.L., Zhao Z.J., Blick K.E., Dooley W.C., Ding W.Q. (2016). Plasma exosome micrornas are indicative of breast cancer. Breast Cancer Res..

[30] Shimomura A., Shiino S., Kawauchi J., Takizawa S., Sakamoto H., Matsuzaki J., Ono M., Takeshita F., Niida S., Shimizu C. (2016). Novel combination of serum microrna for detecting breast cancer in the early stage. Cancer Sci..

[31] Fu L., Li Z., Zhu J., Wang P., Fan G., Dai Y., Zheng Z., Liu Y. (2016). Serum expression levels of microrna-382-3p, -598-3p, -1246 and -184 in breast cancer patients. Oncol. Lett..

[32] Li S., Yang X., Yang J., Zhen J., Zhang D. (2016). Serum microrna-21 as a potential diagnostic biomarker for breast cancer: A systematic review and meta-analysis. Clin. Exp. Med..

[33] Zhong S., Chen X., Wang D., Zhang X., Shen H., Yang S., Lv M., Tang J., Zhao J. (2016). Microrna expression profiles of drug-resistance breast cancer cells and their exosomes. Oncotarget.

[34] Mihelich B.L., Dambal S., Lin S., Nonn L. (2016). miR-182, of the miR-183 cluster family, is packaged in exosomes and is detected in human exosomes from serum, breast cells and prostate cells. Oncol. Lett..

[35] Wei Z., Batagov A.O., Carter D.R., Krichevsky A.M. (2016). Fetal bovine serum rna interferes with the cell culture derived extracellular rna. Sci. Rep..

[36] Bovy N., Blomme B., Freres P., Dederen S., Nivelles O., Lion M., Carnet O., Martial J.A., Noel A., Thiry M. (2015). Endothelial exosomes contribute to the antitumor response during breast cancer neoadjuvant chemotherapy via microrna transfer. Oncotarget.

[37] Wei Y., Lai X., Yu S., Chen S., Ma Y., Zhang Y., Li H., Zhu X., Yao L., Zhang J. (2014). Exosomal miR-221/222 enhances tamoxifen resistance in recipient er-positive breast cancer cells. Breast Cancer Res. Treat..

[38] Chen W.X., Liu X.M., Lv M.M., Chen L., Zhao J.H., Zhong S.L., Ji M.H., Hu Q., Luo Z., Wu J.Z. (2014). Exosomes from drug-resistant breast cancer cells transmit chemoresistance by a horizontal transfer of micrornas. PLoS ONE.

[39] Zhong S., Li W., Chen Z., Xu J., Zhao J. (2013). miR-222 and miR-29a contribute to the drug-resistance of breast cancer cells. Gene.

[40] Mao L., Li J., Chen W.X., Cai Y.Q., Yu D.D., Zhong S.L., Zhao J.H., Zhou J.W., Tang J.H. (2016). Exosomes decrease sensitivity of breast cancer cells to adriamycin by delivering micrornas. Tumour Biol..

[41] Shen H., Li L., Yang S., Wang D., Zhong S., Zhao J., Tang J. (2016). Microrna-29a contributes to drug-resistance of breast cancer cells to adriamycin through pten/akt/gsk3beta signaling pathway. Gene.

[42] Shen H., Wang D., Li L., Yang S., Chen X., Zhou S., Zhong S., Zhao J., Tang J. (2017). Mir-222 promotes drug-resistance of breast cancer cells to adriamycin via modulation of pten/akt/foxo1 pathway. Gene.

[43] Yu D.D., Wu Y., Zhang X.H., Lv M.M., Chen W.X., Chen X., Yang S.J., Shen H., Zhong S.L., Tang J.H. (2016). Exosomes from adriamycin-resistant breast cancer cells transmit drug resistance partly by delivering miR-222. Tumour Biol..

[44] Wei Y., Li M., Cui S., Wang D., Zhang C.Y., Zen K., Li L. (2016). Shikonin inhibits the proliferation of human breast cancer cells by reducing tumor-derived exosomes. Molecules.

[45] Donnarumma E., Fiore D., Nappa M., Roscigno G., Adamo A., Iaboni M., Russo V., Affinito A., Puoti I., Quintavalle C. (2017). Cancer-associated fibroblasts release exosomal micrornas that dictate an aggressive phenotype in breast cancer. Oncotarget.

[46] Gernapudi R., Yao Y., Zhang Y., Wolfson B., Roy S., Duru N., Eades G., Yang P., Zhou Q. (2015). Targeting exosomes from preadipocytes inhibits preadipocyte to cancer stem cell signaling in early-stage breast cancer. Breast Cancer Res. Treat..

[47] O’Brien K., Lowry M.C., Corcoran C., Martinez V.G., Daly M., Rani S., Gallagher W.M., Radomski M.W., MacLeod R.A., O’Driscoll L. (2015). Mir-134 in extracellular vesicles reduces triple-negative breast cancer aggression and increases drug sensitivity. Oncotarget.

[48] Singh R., Pochampally R., Watabe K., Lu Z., Mo Y.Y. (2014). Exosome-mediated transfer of mir-10b promotes cell invasion in breast cancer. Mol. Cancer.

[49] Le M.T., Hamar P., Guo C., Basar E., Perdigao-Henriques R., Balaj L., Lieberman J. (2014). Mir-200-containing extracellular vesicles promote breast cancer cell metastasis. J. Clin. Investig..

[50] Baroni S., Romero-Cordoba S., Plantamura I., Dugo M., D’Ippolito E., Cataldo A., Cosentino G., Angeloni V., Rossini A., Daidone M.G. (2016). Exosome-mediated delivery of mir-9 induces cancer-associated fibroblast-like properties in human breast fibroblasts. Cell Death Dis..

[51] Fong M.Y., Zhou W., Liu L., Alontaga A.Y., Chandra M., Ashby J., Chow A., O’Connor S.T., Li S., Chin A.R. (2015). Breast-cancer-secreted mir-122 reprograms glucose metabolism in premetastatic niche to promote metastasis. Nat. Cell Biol..

[52] Zhou W., Fong M.Y., Min Y., Somlo G., Liu L., Palomares M.R., Yu Y., Chow A., O’Connor S.T., Chin A.R. (2014). Cancer-secreted mir-105 destroys vascular endothelial barriers to promote metastasis. Cancer Cell.

[53] Tominaga N., Kosaka N., Ono M., Katsuda T., Yoshioka Y., Tamura K., Lotvall J., Nakagama H., Ochiya T. (2015). Brain metastatic cancer cells release microRNA-181c-containing extracellular vesicles capable of destructing blood-brain barrier. Nat. Commun..

[54] Hannafon B.N., Carpenter K.J., Berry W.L., Janknecht R., Dooley W.C., Ding W.Q. (2015). Exosome-mediated microrna signaling from breast cancer cells is altered by the anti-angiogenesis agent docosahexaenoic acid (DHA). Mol. Cancer.

[55] Lee J.K., Park S.R., Jung B.K., Jeon Y.K., Lee Y.S., Kim M.K., Kim Y.G., Jang J.Y., Kim C.W. (2013). Exosomes derived from mesenchymal stem cells suppress angiogenesis by down-regulating vegf expression in breast cancer cells. PLoS ONE.

[56] Ono M., Kosaka N., Tominaga N., Yoshioka Y., Takeshita F., Takahashi R.U., Yoshida M., Tsuda H., Tamura K., Ochiya T. (2014). Exosomes from bone marrow mesenchymal stem cells contain a microrna that promotes dormancy in metastatic breast cancer cells. Sci. Signal..

[57] Lim P.K., Bliss S.A., Patel S.A., Taborga M., Dave M.A., Gregory L.A., Greco S.J., Bryan M., Patel P.S., Rameshwar P. (2011). Gap junction-mediated import of microrna from bone marrow stromal cells can elicit cell cycle quiescence in breast cancer cells. Cancer Res..

[58] Bliss S.A., Sinha G., Sandiford O.A., Williams L.M., Engelberth D.J., Guiro K., Isenalumhe L.L., Greco S.J., Ayer S., Bryan M. (2016). Mesenchymal stem cell-derived exosomes stimulate cycling quiescence and early breast cancer dormancy in bone marrow. Cancer Res..

[59] Guire V., Fabbri M., Tsongalis G.J. (2017). Not all good things come in big packages. Clin. Chem. Lab. Med..

[60] Ostenfeld M.S., Jensen S.G., Jeppesen D.K., Christensen L.L., Thorsen S.B., Stenvang J., Hvam M.L., Thomsen A., Mouritzen P., Rasmussen M.H. (2016). Mirna profiling of circulating epcam(+) extracellular vesicles: Promising biomarkers of colorectal cancer. J. Extracell. Vesicles.

[61] Rupp A.K., Rupp C., Keller S., Brase J.C., Ehehalt R., Fogel M., Moldenhauer G., Marme F., Sultmann H., Altevogt P. (2011). Loss of epcam expression in breast cancer derived serum exosomes: Role of proteolytic cleavage. Gynecol. Oncol..

[62] Fabbri M., Paone A., Calore F., Galli R., Gaudio E., Santhanam R., Lovat F., Fadda P., Mao C., Nuovo G.J. (2012). Micrornas bind to toll-like receptors to induce prometastatic inflammatory response. Proc. Natl. Acad. Sci. USA.

[63] Challagundla K.B., Wise P.M., Neviani P., Chava H., Murtadha M., Xu T., Kennedy R., Ivan C., Zhang X., Vannini I. (2015). Exosome-mediated transfer of micrornas within the tumor microenvironment and neuroblastoma resistance to chemotherapy. J. Natl. Cancer Inst..

[64] Fabbri M. (2012). Tlrs as mirna receptors. Cancer Res..

[65] Corcoran C., Friel A.M., Duffy M.J., Crown J., O’Driscoll L. (2011). Intracellular and extracellular micrornas in breast cancer. Clin. Chem..

[66] Melo S.A., Sugimoto H., O’Connell J.T., Kato N., Villanueva A., Vidal A., Qiu L., Vitkin E., Perelman L.T., Melo C.A. (2014). Cancer exosomes perform cell-independent microrna biogenesis and promote tumorigenesis. Cancer Cell.

[67] Alamolhodaei N.S., Behravan J., Mosaffa F., Karimi G. (2016). Mir 221/222 as new players in tamoxifen resistance. Curr. Pharm. Des..

[68] Egeland N.G., Lunde S., Jonsdottir K., Lende T.H., Cronin-Fenton D., Gilje B., Janssen E.A., Soiland H. (2015). The role of micrornas as predictors of response to tamoxifen treatment in breast cancer patients. Int. J. Mol. Sci..

[69] Santos J.C., Ribeiro M.L., Sarian L.O., Ortega M.M., Derchain S.F. (2016). Exosomes-mediate micrornas transfer in breast cancer chemoresistance regulation. Am. J. Cancer Res..

[70] Yu S., Wei Y., Xu Y., Zhang Y., Li J., Zhang J. (2016). Extracellular vesicles in breast cancer drug resistance and their clinical application. Tumour Biol..

[71] Chen W.X., Cai Y.Q., Lv M.M., Chen L., Zhong S.L., Ma T.F., Zhao J.H., Tang J.H. (2014). Exosomes from docetaxel-resistant breast cancer cells alter chemosensitivity by delivering micrornas. Tumour Biol..

[72] Fischer K.R., Durrans A., Lee S., Sheng J., Li F., Wong S.T., Choi H., El Rayes T., Ryu S., Troeger J. (2015). Epithelial-to-mesenchymal transition is not required for lung metastasis but contributes to chemoresistance. Nature.

[73] Ma L., Reinhardt F., Pan E., Soutschek J., Bhat B., Marcusson E.G., Teruya-Feldstein J., Bell G.W., Weinberg R.A. (2010). Therapeutic silencing of mir-10b inhibits metastasis in a mouse mammary tumor model. Nat. Biotechnol..

[74] Chevillet J.R., Kang Q., Ruf I.K., Briggs H.A., Vojtech L.N., Hughes S.M., Cheng H.H., Arroyo J.D., Meredith E.K., Gallichotte E.N. (2014). Quantitative and stoichiometric analysis of the microrna content of exosomes. Proc. Natl. Acad. Sci. USA.

[75] Zomer A., Maynard C., Verweij F.J., Kamermans A., Schafer R., Beerling E., Schiffelers R.M., de Wit E., Berenguer J., Ellenbroek S.I. (2015). In vivo imaging reveals extracellular vesicle-mediated phenocopying of metastatic behavior. Cell.

[76] Squadrito M.L., Baer C., Burdet F., Maderna C., Gilfillan G.D., Lyle R., Ibberson M., De Palma M. (2014). Endogenous rnas modulate microrna sorting to exosomes and transfer to acceptor cells. Cell Rep..

[77] Hoshino A., Costa-Silva B., Shen T.L., Rodrigues G., Hashimoto A., Tesic Mark M., Molina H., Kohsaka S., Di Giannatale A., Ceder S. (2015). Tumour exosome integrins determine organotropic metastasis. Nature.

